# Using Online Support Communities for Tourette Syndrome and Tic Disorders: Online Survey of Users’ Experiences

**DOI:** 10.2196/18099

**Published:** 2020-11-03

**Authors:** Victoria Perkins, Neil S Coulson, E Bethan Davies

**Affiliations:** 1 Division of Rehabilitation, Ageing and Wellbeing School of Medicine University of Nottingham Medical School, Queen's Medical Centre Nottingham United Kingdom; 2 Division of Psychiatry & Applied Psychology Institute of Mental Health The University of Nottingham Nottingham United Kingdom; 3 NIHR MindTech MedTech Co-operative, Institute of Mental Health The University of Nottingham Nottingham United Kingdom

**Keywords:** social network, social support, qualitative research, online community, online support group, Tourette syndrome, tic disorders

## Abstract

**Background:**

People living with a tic disorder (TD)—such as Tourette syndrome (TS)—experience many negative psychological and social challenges arising from chronic tics, such as stigmatization from peers and poorer quality of life, and these can impact upon their families too. It can be difficult for this population to access face-to-face support for tics, and so online support communities offer one avenue for support from peers facing similar experiences. However, little is known about how online support communities may be used by people with TS and other TDs, and by others (eg, parents, caregivers) supporting a person with TS/TD.

**Objective:**

This study aimed to explore users’ experiences of participation in online support communities for TS and TDs.

**Methods:**

In total, 90 respondents (aged 13-62 years; 62% [56/90] female) from 13 countries completed an online survey exploring their experiences of using online support communities for TS and TDs. Respondents were people living with TS/TD themselves (n=68) or supportive others of someone with TS/TD (eg, parent, sibling, spouse; n=14), or both (n=8). The online survey contained open-ended questions eliciting their self-reported motivations for using online communities, their benefits and drawbacks of participation, and whether online support communities affected offline management of tics. Responses were analyzed using thematic analysis.

**Results:**

Seven overarching themes captured experiences of using online support communities for TS/TDs. The overwhelming reason for their use was to find accessible support due to a lack of offline face-to-face support. Online support communities were valued sources of informational and emotional support, and also had a positive impact upon helping users’ psychological well-being. Online communities helped provide a space where people with TS/TDs could feel accepted and reduce the social isolation they felt offline. The suggestible nature of tics and being reminded of the challenging nature of TDs were main disadvantages arising from using online support communities, alongside conflict arising within online communities.

**Conclusions:**

The findings suggest that online support communities appear to offer valuable informational and emotional support to those living with TS/TD and their families too, especially given the lack of locally available support. This facilitates a sense of community online, which can help users in overcoming long-standing social isolation and aid self-reported improvements in psychosocial well-being. Users reported some drawbacks in engaging with online support communities, such as conflict between different types of users and triggering content, which negatively affected experiences of community participation.

## Introduction

Tic disorders (TDs)—such as Tourette syndrome (TS)—are noncurable neurodevelopmental conditions characterized by sudden, persistent, purposeless motor movements, or vocalizations known as tics [1]. TDs typically have their onset during childhood, and tend to peak in early adolescence [2]. Transient TD (ie, tics present for <1 year) is the most common, affecting approximately 3% of children; chronic TD (motor or vocal tics present for >1 year) and TS (motor and vocal tics for >1 year) affect approximately 1.61% and 0.77%, respectively [3]. Tics fluctuate in both frequency and severity over time, and for many tend to decline with age [4].

The processes involved in the assessment, diagnosis, and treatment of TDs can be tricky to navigate: delays in referral and diagnosis are common due to the fluctuating nature of tics, and how they can be indicative of other health issues (eg, eye problems, allergies) [5]. Patients with TDs describe many health care professionals as being uninformed about tics, in turn contributing to delays in diagnosis and intervention [6]. Furthermore, environmental and systemic barriers—such as geographical location, issues with funding, and service delays—between specialist services and patients often hinder access [7,8]. Evidence-based pharmacological and behavioral treatments and supportive care strategies can facilitate reductions in tics [9,10]. Pharmacological and behavioral treatments are effective for TDs but with unique issues affecting their use: for example, medications are often accompanied by adverse effects (eg, fatigue, drowsiness, weight gain) [7,11]. Additionally, TDs are seldom seen in isolation; attention deficit hyperactivity disorder, obsessive-compulsive disorder, autism spectrum disorder, and learning disorders are common comorbidities [12,13]. These comorbidities can further affect assessment and treatment options, as well as further impacting upon quality of life (QoL).

Chronic tics can impair functioning across many areas of everyday life for children and adults [2]. This includes their physical impact (eg, pain and injury from tics), emotional and psychological impact (eg, increased anxiety and hopelessness, questioning sense of self), impact upon school and work (eg, tics impacting upon classroom learning, misunderstanding from teachers, finding employment), and impact upon social life and relationships (eg, bullying, affecting family dynamics) [2,14,15]. TDs also impact upon the family too: interviews with parents of children with TS describe having to cope daily with tic-related challenges, such as seeing their child have painful tics, disruption to daily routines, and financial difficulties arising from additional caring responsibilities and tic-related behaviors [16]. The visible nature of tics often attracts attention from other people [17], and the negative impact of chronic tics on social relationships is widely known: stigmatization from others, discrimination, isolation, bullying, and social exclusion arising from TDs are common interpersonal issues found across the life span [6,18]. The consequences of childhood rejection and stigma can be long-lasting, with adults in one study describing purposely avoiding others to evade ridicule [18].

Social support is an important resource in coping with chronic conditions [19], and may help lessen the detrimental impact of TDs upon psychosocial well-being of individuals with TD and their families [20]. Receiving social support from others with similar lived experiences can help reduce isolation and aide adjustment and coping with tics [16,18,21]. Wadman et al [17] report how young people may find it difficult talking to their non-TS peers about tics—for example, being concerned about others’ reactions and perceptions—and suggest learning about other strategies people with TS use to educate others could be beneficial. Social support is important for families too: in an Australian study involving mothers of children with TS, respondents described the social isolation and decline in social support arising from the challenges of TS, and placed particular importance on the value of making connections with people who understood the lived experience of TS—such as other parents [21]. Furthermore, increased perceived social support among parents of children with TD was associated with emotional and practical coping mechanisms that helped manage tic-related difficulties [20]. However the relative rarity of TDs means local support—particularly for adults with TD—is seldom accessible [18].

With increasing access to the internet, online support communities provide new opportunities for people affected by specific health issues to share, communicate, and network together in an online space [22,23]. Online support offers many features that are not available in face-to-face offline support, including reducing geographical and financial restrictions in access, greater anonymity in participation, and being able to access informational and emotional support that may not be available offline [22,24]. The content of online support communities is generated by the users themselves, meaning they can discuss issues important to them. Online support communities are typically asynchronous platforms (eg, online forums on charity websites) and in recent years disease-specific groups have emerged on social networking sites (eg, Facebook) [23,25]. Furthermore, participation in online support communities can be more *active* or *passive*: users may actively engage in the community (eg, post messages, respond to others, upvote/like others’ content) or be more passive in reading others’ content but not interacting with it [26]. Benefits and drawbacks of online support community participation may vary by the type of chronic illness support is sought for; for example, women using a polycystic ovary syndrome online support community reported feeling empowered by reading about others living fulfilled lives despite their condition [27], while users of an inflammatory bowel disease online support community found peer support useful in guiding their treatment decisions [28]. For young people, online support communities can help validate and normalize what they are experiencing, sharing their thoughts, feelings, and experiences with similar others [24]. Online support communities are not without their problems and a range of difficulties have been described in the literature: greater anonymity and distance mean users may be rude to others, the wealth of information can be difficult to process and inaccurate information can be shared without fact checking, and the absence of facial and vocal cues in online communication can lead to misunderstandings in support [29]. However, whether there exists any problems arising from engagement with TD online support communities remains to be seen.

For individuals with TDs and their support networks, online support communities could potentially provide a viable outlet to find social support from peers facing similar tic-related challenges, which subsequently may facilitate management of tics and aid their psychosocial well-being. People with TS have described how they may conceal and suppress their tics in public [17,18,30]: people with TS may have more control over how they present themselves online, and tics (and common comorbidities) may interfere less in seeking out online support compared with face-to-face. Previous research has highlighted the importance of peer acceptance and supportive friendships in providing practical and emotional support [6], but to date there has been little exploration of social support from peers with TDs. Therefore the aim of this study is to explore users’ perceptions and experiences of using online support communities for TDs, in order to gain insight into their participation in online support communities, benefits and drawbacks of being using online support communities, and the impact of using online support communities upon their offline management of TDs. Users of these online support communities reflect those with a TD (diagnosed or suspected), and supportive others (eg, parents, siblings, spouses and friends).

## Methods

### Participants and Recruitment

The study involved recruiting users of online support communities for TDs and TS. Relevant online support communities were identified via searches using Google and on Facebook employing a range of key terms in different combinations, such as *online support*, *online group* and *tics*, *tic disorders*, or *Tourette syndrome*. As there had been previous little research exploring online support communities for TDs/TS, it was felt that the criteria outlined by Coulson et al [23] provided a methodical way to identify suitable and active online support communities for this study. To identify eligible online support communities, they had to be (1) user led (ie, not led by health professionals) and *currently active*, meaning they had a minimum of 30 postings per month; (2) in English language; and (3) the terms and conditions of the online support communities did not preclude research. In online support communities where administrators/moderators were identifiable and contactable, an introductory message was sent to them requesting permission to post study information in the online support community. Where no administrators/moderators were apparent, the lead researcher (VP) independently posted the study advert directly to the online support community. Together, 14 eligible online support communities were contacted for recruitment purposes (n=11 Facebook groups, n=3 forums on other websites). Permission to recruit was obtained from 4 online support communities, with the remaining either declining to participate (n=7; reasons for declining were not provided) or failing to respond (n=3). Additionally, the advert was circulated by Tourettes Action (a UK-based TS charity) via their newsletter and social media channels, and was shared via Twitter by the NIHR MindTech MedTech Co-operative.

After identifying eligible online support communities, to take part in the study participants were (1) people with TD/TS themselves (diagnosed or suspected) or be someone supporting a person with TD/TS (eg, a parent, sibling, partner, or friend), or could be in both categories; (2) aged 13 years or older, as is typically the minimum age legally required to register to use online support communities [28]; and (3) be a member of 1 (or more) online support community specifically for TDs.

The study adverts provided information about the study aims and invited individuals to click on a link to the online survey (hosted on Jisc Online Surveys). On the survey’s landing page, respondents were presented with a more comprehensive overview of the study and their legal rights and protections. Participants were required to confirm they had read and understood the information and indicated their willingness to participate through completing an online consent form. Next, participants completed questions about themselves, their lived experience of TD/TS, and self-reported use of online support communities, as well as 6 open-ended questions exploring their personal motivations for using online support communities, benefits and drawbacks of participation, and ways in which the online support had impacted upon offline management of TDs (Textbox 1). The questions were based on those from previous research exploring online support community use for different health issues and chronic conditions [23,27,28,31,32]. Each question was accompanied by a free-text box, providing participants the opportunity to share their experiences.

**Open-ended questions presented in the online survey.**

1. What first led you to use an online community for Tourette syndrome/tics?
2. What, if any, do you consider to be the main benefits to participating in online communities for Tourette syndrome and/or tics?
3. What, if any, drawbacks have you encountered in participating in online communities for Tourette syndrome and/or tics?
4. How have online communities influenced the way you manage your tics, or how you help the person you support (child/sibling/spouse/friend) with their tics? If so, please give some examples.
5. Has anything you have been made aware of or learnt from online communities helped you or the person you support (child/sibling/spouse/friend) in any other way? If so, please give some examples.
6. Is there anything else you wish to tell us about your experience of using online communities for Tourette syndrome and/or tics?

### Patient and Public Involvement

Prior to finalizing the 6 open-ended questions, potential questions were discussed with a member of the Patient and Public Involvement (PPI) group from the Online Remote Behavioural Interventions for Tics trial (ORBIT trial [33]): the PPI group members have a diagnosis of TS or are a parent to a child with TS. Topics arising from this conversation were used to structure the final set of questions. The full online survey was subsequently reviewed by 3 members of the PPI panel to check it was understandable, appropriate, and sensitive to its intended audience.

### Ethical Considerations

The study was reviewed and approved by the University of Nottingham Division of Rehabilitation, Ageing & Wellbeing Ethics Committee. The first 2 pages of the online survey consisted of comprehensive study information, rights in participation, and withdrawal procedures, alongside the research team’s contact details. The online consent form consisted of several statements, all of which the participant needed to agree to in order to participate in the study. All responses were kept securely: access to data was granted to members of the research team only, and all data were downloaded to and stored on a password-protected computer. No participants chose to withdraw after completing the online survey.

### Analysis

Responses to the open-ended questions were analyzed by the first author (VP), using an inductive approach to thematic analysis [34]. As the role of online support communities for TDs had not been explored previously in research, a data-driven inductive approach without a theoretically derived coding scheme was judged as a practical analytic strategy to explore all aspects of the data. Analysis followed Braun and Clarke’s [34] guidelines: first, data familiarization and searching for initial meanings were performed through reading and re-reading each survey response several times. Second, preliminary codes detailing basic interpretation of responses were made, and similarly coded data were grouped together. Next, similarly coded data were organized into related clusters, which generated initial theme names. These initial themes were reviewed and the data used to support them examined. In instances where evidence gathered was insufficient to support a theme, codes were regrouped. Themes were defined and refined into subthemes to ensure findings credibly captured all important elements of the data set. o support this process, the second (NC) and third (EBD) authors reviewed the themes/subthemes and coded data, and with the first author (VP) finalized the themes.

## Results

### Participant Demographics

A total of 90 participants completed the online survey (Table 1): the majority (68/90, 76%) had a TD themselves. Slightly less than two-thirds of the sample were female (56/90, 62%), with ages ranging from 13 to 62 years (mean 27.28 [SD 13.11] years), and the majority resided in the United States (38/90). Participants varied in how long they (or the person they supported) had been living with tics, ranging from 6 months to 50 years (mean 12.56 [SD 13.19] years; median 9 years). Self-reported comorbidities were common, with over four-fifths (74/90, 82%) reporting at least one comorbid condition. The majority were members of more than 1 online support community (68/90, 76%) and reported using them for less than a year (53/90, 59%). Many respondents self-reported accessing online support communities frequently, with one-third accessing online support communities everyday (32/90) and another third accessing several times a week (34/90).

Thematic analysis generated 7 overarching themes reflecting reasons for accessing online support, the informational and emotional support gained from online support communities, feeling accepted and reducing isolation in having TDs, and problems with using online support.

**Table 1:** Demographic composition of sample (N=90).

Demographic		Value
**Gender, n (%)**		
	Male	25 (28)
	Female	56 (62)
	Nonbinary	6 (7)
	Other	2 (2)
**Age, mean (SD)**		
	Person with TD^a^ (n=68)	24.04 (11.32)
	Person with TD who also supports someone with TD (n=8)	31.38 (16.07)
	Non-TD person who supports someone with TD (n=14)	40.64 (10.93)
**Location, n (%)**		
	Australia	8 (9)
	Austria	1 (1)
	Belgium	3 (3)
	Canada	5 (6)
	Germany	5 (6)
	Ireland (Republic of)	1 (1)
	Italy	2 (2)
	Netherlands	1 (1)
	New Zealand	1 (1)
	Norway	1 (1)
	Poland	1 (1)
	United Kingdom	23 (26)
	United States	38 (42)
**Self-reported co-occurring conditions^b^, n (%)**		
	Autism spectrum disorder	22 (24)
	Attention deficit hyperactivity disorder	27 (30.0)
	Obsessive-compulsive disorder	41 (46)
	Anxiety	62 (69)
	Depression	39 (43)
	Dyslexia or other learning difficulty	13 (14)
	Dyspraxia	6 (7)
	Other	13 (14)
**Membership of TS^c^/TD online support communities, n (%)**		
	Member of one online support community	21 (23)
	Member of two or more online support communities	69 (77)
**Frequency of using TS/TD online support communities, n (%)**		
	Every day	32 (36)
	Several times a week	34 (38)
	Once a week	10 (11)
	Once every couple of weeks	5 (6)
	Once a month	3 (3)
	Once every couple of months	1 (1)
	Once a year	2 (2)
**Length of using online support communities for TS/TD, n (%)**		
	0-6 months	33 (37)
	7-12 months	20 (22)
	1-2 years	12 (13)
	More than 2 years	25 (28)

^a^TD: tic disorder.

^b^Values add up to more than 100% as participants could self-report more than one co-occurring condition.

^c^TS: Tourette syndrome.

### Using the Internet to Find Accessible Support

This theme describes how minimal availability of face-to-face support encouraged participants to seek out support from peers online.

#### Lack of Offline Support From Peers and Health Care Practitioners

Participants discussed how lack of in-person support—from people with TD and from health professionals—for TDs motivated their use of online platforms to find support.

I want to interact with others who also have the condition (...) adults have so few options for support.

P40, aged 23, has TD

Participants described their wish to access support from others experiencing TDs, and their dissatisfaction regarding the limited face-to-face opportunities to do so. Additionally implied is that many health care professionals appeared to have a basic understanding of TDs, but lacked a thorough appreciation of the difficulties faced by their patients. Finding and using online support communities was seen as a viable option to aid this.

Medical professionals need to realise that people go to these forums more than their doctors because your standard GP etc has no idea what TS is really like, we provide our own support because no one else will. We are like a forgotten element of the medical society.

P27, aged 43, has TD

#### Breaking Geographical Barriers

Despite some discontent regarding limited face-to-face support, multiple participants appreciated how online groups meant they could access support regardless of their geographical location to account for their limited face-to-face support from others with TDs:

Such a valuable resource when don’t have any local TS families.

P24, aged 46, parent of child with TD

While face-to-face support groups may provide small local networks for patients, online support communities facilitate cross-cultural interactions providing people greater choice and opportunity to find and communicate with similar peers.

#### Ease of Access

Being able to access support via online support communities at any time was considered beneficial compared with scheduled face-to-face groups, especially by those who felt that accessing face-to-face support was challenging:

I actually prefer the online connections as opposed to in person because I have anxiety and the internet makes it easier for me to talk to people.

P44, aged 19, has TD

A small number of participants also advocated for online communication as helpful when comorbid conditions hindered their attendance at face-to-face groups.

### Online Communities as Informational Sources

This theme explores how information obtained in online support communities supports users’ offline management of TDs and common comorbidities. Experienced online support community users were depicted as *experts* by participants, well-known for providing practical advice and strategies for lessening the impact of TDs.

#### Signposting to Health Services and Diagnostic Advice

Signposting newer members to appropriate health services and charities was commonly reported by participants, and had assisted many in acquiring a TD diagnosis and guidance on ways to explain tics and comorbidities to others. For many participants, the decision to access online support communities was motivated by concerns they may have a TD:

My initial suspicions that I might have TS led me to search for online communities (...) that community convinced me to speak to a doctor.

P11, aged 50, has TD

#### Helpful in Managing Tics

Several participants described how they tried out strategies shared by online support community members, such as learning to *redirect* tics, to manage their tics:

I’ve learned how to shorten my tic attacks, tricks and tips for painful tics like a head pillow for neck jerking and padded gloves for self-hitting tics.

P13, aged 20, has TD

One use of online support communities by participants was to share acquired behavioral and lifestyle management techniques with struggling, newly diagnosed members. Participants described how online support communities can facilitate TD management by allowing users to seek out personalized advice pertaining to specific, bothersome, and often self-injurious tics.

#### Understanding Common Comorbidities to Gain Personal Insight

Information shared regarding common comorbidities appeared to reassure and comfort users (both people with TD and parents supporting a child) about the commonness of comorbidities with and the complexities involved in managing multiple conditions:

The awareness of the comorbidities that come alongside Tourettes has helped me and my family be less confused and worried as to why I had such bad OCD.

P25, aged 16, has TD and sibling with TD

#### Explaining Tics to Other People

Respondents described how reading other users’ accounts of similar discussions aided how they communicated their TD to others, and used information obtained from online support communities to facilitate offline discussion with other people:

I learned a lot of information about Tourette's, a lot of personal accounts that aligned with my experiences and comforted my worries, and ways to handle opening up about my TS to friends and family.

P80, aged 21, has TD

Being signposted to informational resources designed for teachers and classmates was reported to have great benefit in helping schools provide effective support:

It has helped me to find information for school on how to deal with tics and about how Tourette’s symptoms manifest themselves.

P31, aged 53, parent of child with TD

#### Patients Are Experts by Experience

Participants were keen to highlight the importance of acquiring information from people with lived experience of TD:

Support & advice from people & other families who have the experience of living with TS.

P24, aged 46, parent of child with TD

On occasion peer expertise was discussed by participants somewhat evocatively, denoted using capital letters or emotive language to emphasize the conviction underpinning the sentiment. This seemingly reflected a perceived need to accentuate the invaluable nature of patient voice in developing a nuanced understanding of TDs.

### Online Communities as Emotional Support Sources

Respondents appreciated online support communities for providing an outlet for sharing and discussion between those experiencing similar difficulties. Mutuality was considered fundamental for other members to gain a comprehensive understanding of personal TD experiences. Such insight fostered emotional support and cultivated online environments in which participants felt comfortable in speaking openly and able to build friendships with likeminded users.

#### Seeking Understanding Through Shared Experiences

Narratives of lived experiences were felt to convey more valuable insights into aspects of TDs that informational sources could not offer. People with TDs were considered to possess a much-desired level of empathy and relatedness that those in participants’ offline world (eg, parents, siblings, non-TD friends) could not provide:

Contact with others who know what it's like to live with TS, WE are the real experts.

P27, aged 43, has TD

#### Sharing in a Safe and Supportive Space

People with TD and those supporting them reported that online support communities provided them the opportunity to express feelings freely and safely:

It can help you get what you’re feeling out. If you’re stressed about something or need to rant you can.

P19, aged 16, has TD

Inferred was that online support communities provide outlets for members to discuss ongoing difficulties unreservedly and without the *censorship* they may experience when discussing emotive events with people in their offline world. The internet-mediated nature of communication between online support community members seemingly allowed participants to speak honestly and receive unquestioning empathy and support without fear of ramification.

#### Forging Friendships and Bonding With Peers

Through receiving emotional support from likeminded others and communicating with people who understood the lived experience of TD, participants detailed how online support communities helped support the development of new friendships:

These communities are a great place to find (...) friends who you can talk and play games with and support that extends beyond tics into general life problems.

P13, aged 20, has TD

### Feeling Part of a Community

Participation in supportive online support communities, particularly smaller communities, reportedly evoked a sense of community among members. The bonds formed with peers in online support communities were considered to replicate in-person support networks and facilitated connectedness and belonging to online environments.

#### Reducing a Sense of Social Isolation

People with TD and those supporting them both felt online support communities helped them feel less removed from society. Interacting with online support community members helped reduce feelings of loneliness, and many reported feeling less *abnormal* or *weird* as a result of using online support communities:

Until I found the community, I was more or less convinced that I was alone in the way I felt – often rejected and embarrassed about my tics. I don’t feel like that anymore.

P10, aged 21, has TD

For some, contact with individuals experiencing TDs was sufficient to feel integrated into a community, while others were inducted by reading members’ TD experiences and drawing parallels to personal life events.

#### Acceptance and Belonging

Participants discussed being immersed in accommodating, nonjudgmental online environments, with closeness and camaraderie between group members facilitating a sense of belonging:

A wonderful [OSC] for Tourette’s has helped so many people with accepting not only themselves but others and has made everyone a close family.

P34, aged 17, has TD

Connections between online support community members were depicted as akin to familial relationships, further illustrating the community atmosphere cultivated within online support communities.

#### Paying It Forward

Several participants reported that sharing their experiences and expertise were useful to newer, struggling online support community members. Sharing experiences to engage in a process of reciprocal support was commonly discussed:

If people are going through the same struggles I did when I was younger I want to offer advice.

P84, aged 21, has TD

Participants described a desire to *give back* to communities who had helped them throughout difficult times and a sense of validation of their abilities to support others. This seemingly perpetuated a cycle of reciprocal support that enabled better adjusted participants to remain connected to the group and in continued receipt of other psychosocial benefits.

#### Preference for Smaller Community

Some participants reported it being more difficult to find a sense of community and community benefits (friendship, closeness, and belonging) in larger online support communities:

It is hard to develop strong friendships or have conversations within groups of hundreds of members.

P82, aged 17, has TD

For many, the inability to engage with all online support community members was a drawback and some participants expressed concerns regarding the motivations of lesser known, often silent members. Overall, respondents typically felt more comfortable in and supported by smaller, intimate online support communities.

### Positive Impact on Psychosocial Well-Being

This theme denotes the positive impact online support communities have on participants’ attitudes and behaviors toward tics. Learning of others’ TD experiences resulted in a shift in beliefs about tics; several respondents expressed gratitude that their tics were less functionally impairing than many users, while some benefitted from the normalizing impact had by witnessing others live fulfilled lives despite tics. In turn this impacted participants’ *offline* world, with many reporting increased social activity and improved self-confidence in the ability to withstand public responses to tics.

#### Reappraising Own Circumstances

Comparison of oneself with other group members was found beneficial to most participants; hearing about more impairing TD experiences seemingly aided participants in reappraising their perception of their own TD, often as being minor compared with others:

Some people struggle with more severe/less socially acceptable tics, such as coprolalia or complex motor tics, I feel like mine aren’t such a big deal after all.

P81, aged 20, has TD

This awareness appeared to help respondents accept their tics as less bothersome or apparent to other people, and reportedly helped minimize self-consciousness about their tics.

#### Improved Confidence and Acceptance of Tics

Through encouraging respondents’ acceptance of tics, online support communities were found to facilitate engagement in typical everyday activities (eg, work, going out):

I’ve learned that I shouldn't be so self-conscious when I tic, that if people ask just explain and move on.

P35, aged 14, has TD

Reducing shame and embarrassment about tics appeared to facilitate their acceptance of them. This revised outlook seemingly bolstered participants’ confidence, enabling many to embrace opportunities to socialize and address their disorder directly in their offline world. Acceptance of tics further aided participants to develop their own personal narratives to explain their tics to others:

They have made me more confident to tic in public. For most of my life, I have suppressed tics in public, leading to the feeling that I am hiding a part of me that I am ashamed of, followed by tic attacks when I get home.

P40, aged 23, has TD

#### Normalization of TDs Provides Hope for the Future

Members with positive lived experiences were found to reassure participants that TDs do not necessarily impact upon long-term QoL:

Seeing other people with TS who are happy, successful and empowered helped me to embrace my TS as it showed me that my condition should give me strength and not hinder me.

P22, aged 16, has TD

Experienced and potentially well-adjusted online support community users were depicted as role models for younger members, capable of offering hope regarding the future by illustrating that patients with TD can achieve the same life goals as typically developing peers. Virtually meeting other people with TD living *normal* lives was depicted as inspiring and encouraged participants to grow and learn from challenging experiences to achieve positive futures.

#### Reduction in Self-Blame

Self-blame was denoted among a handful of predominantly younger online support community members, many who once viewed tics as a *punishment*. Online support communities were found to help overcome this perception: the mechanisms through which this occurred were not discussed, but other positive influences on psychosocial well-being (acceptance, normalization, and reappraisal of circumstances) occurring following exposure to other individuals with TD plausibly helped facilitate this reduced self-blame:

I don’t feel that my tics are my fault. I am trying to convince myself that I’m not doing something wrong by having tics, and this community is helping.

P10, aged 21, has TD

### Problems With Online Support

Both tic-related and internet-based problems with online support community access were described by participants. A frequently mentioned concern by respondents was the tendency for non-evidence–based advice to be discussed and potentially mislead vulnerable users. Another concern centred around posts of graphic tic-related injury, with participants describing how this appeared to lead to an uncontrollable influx of tics, which often compelled temporary withdrawal from online support communities. Furthermore, reading others’ negative experiences perpetuated low mood and discouraged participants’ optimism for the future.

#### Tics Are Suggestible

Online support communities were commonly described as *triggering*, meaning how reading about tics or watching videos of tics could lead to heightened tic severity, frequency, or development of new tics. Most considered this a minor disadvantage, advising that avoiding online support communities when tics are particularly bothersome is a suitable solution:

Sometimes you can pick up new tics, or reading about others will make your own tics increase in frequency. (...) if I am having a “bad tic day” I will not read the posts in the community.

P14, aged 18, has TD

Several participants suggested using *warnings* preceding the body of particularly descriptive or visual posts.

#### Continuous Reminders of a Lifetime of Challenges

Exposure to others’ daily struggles reminded participants that managing TD was a necessary part of life. Negative experiences were discussed somewhat apathetically, with a handful of participants seemingly resigned to a potentially restricted future and accepting difficulties they/the person they support may someday encounter. For some this perpetuated low mood and enhanced the perceived magnitude of TD-related problems:

It can be upsetting to see and read about the negative experiences some have had and can be depressing to be part of a group when you really just wish your child didn't have this extra issue to cope with.

P56, aged 52, parent of child with TD

#### How Sound Is Others’ Advice?

Several participants expressed concerns regarding the validity of advice offered in online support communities:

There are many people who come looking for advice- but a lot of the advice given is misleading, inaccurate and sometimes dangerous.

P58, aged 35, parent of child with TD

Respondents recognized that particularly among parents of children with TD, desperation to improve their child’s QoL led to them being vulnerable to accepting poor advice from unverified sources. Alternative treatment regimens were considered with skepticism, prompting multiple participants to emphasize that online support communities cannot replace medical guidance and recommend others to avoid engaging with experimental or anecdotal therapies.

### Within-Group Conflict

Perceived judgment and a lack of consideration among online support community members were found to foster conflict between users, culminating in a negative community atmosphere. Many participants discussed encounters with unfriendly individuals within online support communities, citing intolerance for personal lifestyle choices and TD management styles as common reasons for withdrawing from online support communities.

#### Unfriendliness of Some Community Members

Respondents felt annoyed by those who expressed entitlement to greater empathy than others; this resulted in somewhat competitive conversations, not conducive to the supportive atmosphere that most members endeavored to create. Participants felt equal respect and acceptance were not afforded to all users:

Some people can be judgemental. In some groups people seem to try to compete for ‘whose Tourette’s is the worst’.

P39, aged 27, has TD

Several participants additionally detailed how insensitive or provocative comments were easily misconstrued and prematurely judged some users. A small number of participants reported experiencing cyber bulling or harassment, contributing to their withdrawal from otherwise supportive networks.

#### Rigidity of Community Perspective

Many recognized that online support communities typically possess a unified and unwavering perception of different approaches for managing tics:

Certain communities tend to develop a common voice. This one strongly supports CBD, that one strongly rejects it. This one loves CBIT, that one is sceptical. (...) either way one isn’t getting a healthy, integrated community or understanding of TS.

P18, aged 38, parent of child with TD and spouse of person with TD

For some, rigid online support community opinions were considered disadvantageous. Several respondents experienced rejection upon initiating discussions regarding topics which did not align with an online support community’s outlook. Additionally, participants commented that newly diagnosed and possibly inexperienced patients with TD may only participate in one online support community, meaning they may only be exposed to certain perspectives or a few resources.

#### Conflict Between Different Types of Users

Frustrations regarding the tendency for parents of younger patients with TD to monopolize groups were common and discussed somewhat evocatively. Conflict between users with a TD and users supporting a person with TD were reported:

I find that parents of children with TS tend to have the loudest voices in some communities. It can focus conversations away from individuals who actually have the condition.

P40, aged 23, has TD

Parents were depicted as insensitive and inconsiderate toward other group users; not only did people with TD feel parent users undervalued their opinions, but many also considered their domineering presence in groups prevented adults with TD from accessing comprehensive social support. Moreover, participants found parents’ use of online support communities as outlets to discuss the negative impact of their child’s TD on family life particularly distressing in questioning their own family’s true feelings toward them.

## Discussion

### Principal Findings

This study explored experiences of online support community users for those living with TDs and people supporting them, such as parents. Seven overarching themes were identified, many of which align with previous research into online support communities for long-term health conditions (eg, [23,27]) and appear to map onto empowering processes and outcomes attained through online communities [35,36], including having increased knowledge about TD, helping shape communication with health professionals, and guiding offline management of tics. Research into social support in TDs has typically looked at support from non-TD peers [17,37], has highlighted the value of support in parents of children with TD [16,21], and investigated social adjustment difficulties in this group [38]. This study adds to the limited literature looking at how people with TD may use online support from peers with TD (or from caregivers of people with TD), and the impact of this upon their tics and well-being.

Our findings suggest several unique benefits of online support communities for TDs. Online groups granted much-desired access to other people with TD, and allowed for diverse cross-cultural connections too. The continuous availability of online support communities allows for support regardless of time boundaries and accessibility to users whose comorbidities deterred attendance to face-to-face support groups. Given the prevalence of morbidities among people with TD [13], this may be particularly useful in recommending online support communities to patients and their families for whom attending in-person groups is particularly psychologically demanding.

Supporting previous literature [6], many respondents felt that medical professionals had inadequate TD knowledge—and so online support communities helped users in gaining personalized guidance and experiential information from fellow peers. This suggests that online support communities can supplement medical advice and offer users sufficient information to manage tics autonomously. Moreover, discussion regarding comorbidities was considered enlightening to participants and provided explanations for long-standing differences between themselves and non-TS peers. This normalization of experiences and sharing of resources appeared to help respondents build their confidence and encouraged initiation of TD-related conversations with others offline (eg, friends, family, teachers). Although online support communities perhaps emphasize the limitations of health care and public TD awareness, their provision of experiential information facilitates better offline management of tics and associated difficulties.

By contrast, 2 drawbacks of online support communities reported by some respondents were the credibility of advice provided by online support community users and rigidity of certain communities in terms of having strong opinions regarding different types of tic management. This could result in users finding it difficult to discuss evidence-based treatments that might not align with the online support community’s preferences, or may be directed toward non-evidence–based treatments. While the role of a moderator/administrator is generally consistent across online support communities (eg, to establish and maintain ground rules through overseeing group communication to maintain safe online space), each community will vary in how they moderate their online space [39], including the discussion of evidence-based and alternative treatments. Voluntary moderators of online support communities are often patients themselves or caregivers of patients [39], and their personal experiences and beliefs about tic management could potentially impact upon how they moderate online support communities. Shoebotham and Coulson [40] suggested that having health professionals as moderators could help in appraising (mis)information posted in online support communities; however, online support communities may wish to be separate from external organizations, and may not want professionals in their patient-led online spaces.

Emotional support was perhaps the greatest incentive for continuing to engage in online support communities for TDs. Aligning with online support communities for other chronic conditions [27,28], respondents benefitted from encountering likeminded others whose mutuality seemingly facilitated a level of understanding not apparent among non-TD networks. This was characterized by distinctions between sympathy and empathy, and provided a foundation for connections otherwise lacking in encounters with non-TD peers. Comfort in being surrounded by people who understood personal tic-related experiences encouraged uncensored and honest discussions, and so respondents depicted online support communities as cathartic outlets in which reassurance, advice, and solidarity were plentiful. This may be in keeping with Cohen & Wills’ [41] direct effect hypothesis: through sharing negative experiences to an online support community, others can provide support, thus rewarding interactions that plausibly shield people with TD (and their supporters) from maladaptive threat appraisals and associated psychosocial impairments [18,38,42].

The culmination of emotional and informational resources seemingly perpetuates supportive online support communities. Many people with TS and families experience rejection in society due to tic-related stigma [43], and exclusion can precipitate social isolation, causing many with TDs to strive for community acceptance [6]. In this study, integration into online support communities was described as a privilege and one which helped reduce the social exclusion experienced offline. Understandably, respondents were protective of the community tone and expressed strong attachments to particularly supportive online support communities. Desire to maintain inclusive communities encouraged many to adopt the supportive role themselves and actively induct newer, inexperienced members. As such, online support communities provide a premade community in which acceptance is not a major struggle.

Meeting other people with TD and comparable difficulties within a group therapy setting may be sufficient enough to improve QoL [44], and several respondents reported online support community use prompted reappraisals of personal circumstances that in turn facilitated positive psychological adjustment (eg, improved confidence, hope for the future, engaging in regular social activities). Smith et al [6] support this, advocating that acceptance of tics into personal identity precipitates quicker adaptation to long-term conditions. Perhaps offering insight into the mechanisms of these psychosocial changes, Cohen and Wills’ [41] buffering hypothesis suggests that by illustrating what people with TD can achieve, supportive others (eg, caregivers) may reframe personal difficulties and help other online support community users from succumbing to maladaptive cognitions which endorse socially isolating behaviors. Additionally, several respondents talked about reappraising the stigma of self-blame and *punishment* they attributed to themselves for having tics. As well as other processes feeding into this reappraisal (eg, acceptance, improved confidence), another factor contributing to this could be through psychoeducational processes within online support communities, such as changing illness representations and new knowledge (eg, understanding more about the neurodevelopmental causes of TDs, the group mentality in online support communities in how they perceive and comprehend TDs). Furthermore, writing about personal experiences in health-specific dedicated online support communities allows users to authentically express themselves and may contribute to sense of empowerment in living with chronic conditions [45]. The findings also suggest online support community members become role models to others, whose adaptive behaviors and attitudes encourage others to participate in typical activities irrespective of tic-related impairments.

Despite the plethora of online support community benefits, participants were forthcoming about a number of limitations. Primarily, the suggestible nature of TDs perpetuated temporary onset of new tics and heightened tic severity upon exposure to graphic posts within online support communities. Similarly, vivid imagery in online support communities can be triggering for people who self-harm [23]. For people with TDs, this appears to be a *double-edged sword* in seeking out support: respondents have described the high value they place in communicating with similar peers, but it comes with risk of experiencing changes in tics (eg, increased frequency, new tics). Respondents in this study felt warnings preceding descriptions or videos of tics would be important in helping other users, and could be enforced by online support community moderators/administrators. Moreover, similar to Holbrey and Coulson [27], a number of members found reading others’ negative TD-related experiences reinforced negative feelings and low mood. Further research is necessary to establish whether this drawback is more prevalent among those with depressive symptomology; however, at present, health care professionals should exert caution when recommending online support communities to people with TD and their families by informing them of the potential risks to their psychological well-being.

As similarly reported for other health issues [23,40,46], conflict between online support community members was a further drawback of engaging in online communities, and should perhaps caution parents of younger community members that online support communities for TD hold similar risks to other internet-based activities. The dominant conflict described by some respondents was resentment among adults with TD toward parents seeking advice regarding their child. Supporting Malli et al [18], adult respondents expressed frustration regarding minimal offline support for adults with TD, and so parental domination of online spaces was felt to further impede access to peer networks. Furthermore, parents’ use of online support communities to discuss the negative impact of a child’s TD endorsed insecurities among adults with TD and concerns regarding familial resentment of them. Having distinct online support communities for people with TDs and their caregivers could be an option to reduce this conflict, but at the same time, there is a balance in that caregivers may learn much from people who have TDs.

### Strengths and Limitations

To our knowledge, this is the first piece of research to examine online support community use for TS/TDs and may provide useful recommendations for practice: online support communities can be proposed as one accessible solution—with cautions—to people with TD and their families otherwise unable to access social support. Moreover, the study positively contributes to a growing literary body pertaining to use of online support communities among those experiencing chronic long-term conditions.

There were several limitations. Although a diverse sample of 90 people of varying ages, genders, and mix of TD-related experiences from across multiple countries participated, they were mostly from Westernized countries and from participants who could respond in English. Future research may wish to explore non-English language online support communities to be more representative. Similarly, the majority of respondents being female was surprising given the higher prevalence of TDs in males [2], and so suggests the acquired sample was not necessarily representative of the prevalence of TD by gender. Furthermore, respondents had not all received a TD diagnosis—with several reporting suspected (but not confirmed) TD—alongside self-reported comorbidities. The nature of the study meant we were unable to confirm whether participants’ suspected TD was diagnosable, and whether comorbidities had been diagnosed too. Additionally, the sample was largely recruited through active online support communities: thus those with negative experiences who discontinued participation were not recruited, plausibly preventing the extent of online support community drawbacks from emerging. Although efforts were made to ask permission from moderators to post the study advert to several online support communities, ultimately 7 declined without explaining their reasons. It may be that patient-led groups wish to stay focused on patient-led discussion and do not want external organizations in their online spaces (eg, due to negative experiences with them; preferences to keep online groups for patients only). Finally, the nature of online survey methodology prevented follow-up questions, which may have provided greater insight and it remains unknown if other unique aspects of online support community use for TDs were not uncovered in this study.

### Conclusion

Overall, online support communities were illustrated as an accessible support source for people with TDs and supportive others. Provision of informational and emotional support was delineated as the foundation for building online support communities, which facilitated a much appreciated sense of inclusion among users, and perpetuated improvements in psychosocial well-being and functioning. As such, despite their drawbacks, online support communities were largely considered hugely beneficial to those experiencing TDs and could be one resource that clinicians may recommend to socially isolated patients and families.

## Abbreviations

CBD: Cannabidiol-based products
CBIT: Comprehensive Behavioural Intervention for Tics
QoL: quality of life
TD(s): Tic disorder(s)
TS: Tourette syndrome
